# Effects of a Chinese herbal extract on the intestinal tract and aquaporin in Adriamycin-induced nephropathy

**DOI:** 10.1080/21655979.2021.2014620

**Published:** 2022-01-22

**Authors:** Weizhong Ma, Xing Liang, Zhuowei Su

**Affiliations:** The Second Clinical College, Guangzhou University of Chinese Medicine, Guangzhou, China

**Keywords:** Aquaporins, radiational Chinese medicine, diarrhea, adriamycin nephropathy

## Abstract

Wuling Decoction is a traditional Chinese medicine that has been used to open knots, benefit water, transform Qi, return fluid, and has a significant effect on strengthening the spleen and removing dampness. To explore the effects of Wuling Decoction on the intestinal tract and aquaporin in Adriamycin-induced nephropathy, 45 specific pathogen free (SPF) Wistar rats were randomly divided into a blank control group (5 rats), Dosing control group (10 rats), Adriamycin nephropathy model group (10 rats), diarrhea group (10 rats), and an Adriamycin nephropathy diarrhea model group (10 rats). The tissue localization of aquaporin (AQP) was determined by immunohistochemistry. The expression of AQP mRNA and protein was measured by RT-PCR and western blot analysis, respectively. The results indicated that Wuling Decoction causes excretion of AQP2 through the urine, regulates AQP2 levels, and exerts diuretic and anti-diarrheal effects. It also regulates the levels of antidiuretic hormone (ADH) and arginine vasopressin (AVP), affects water absorption rate, and reduces the level of cyclic adenosine monophosphate (cAMP) in each tissue, thus reducing the absorption of AQP2 to water. Wuling Decoction promoted AQP2 expression in the nephropathy model group and inhibited AQP2 expression in the diarrhea group. Wuling Decoction increased the expression of aquaporin in the intestinal tract, reduced the water content of stool by promoting the absorption of water in the intestinal tract, inhibited the expression of aquaporin and its regulatory factors in nephridia tissue, and reduced the reabsorption of water to increase urine volume, to decrease the occurrence of diarrhea.

## 1. Introduction

Water is an important component of the human body and plays an indispensable role in all metabolic processes. The occurrence and development of many diseases are associated with an imbalance in water metabolism. The discovery of water channels has facilitated the study of the molecular basis of the water-liquid balance and enabled the discovery of the underlying mechanism and treatment targets of water-liquid imbalance syndromes. Normally, water channel proteins are widely distributed in each organ, tissue, and cell. They belong to a class of membrane proteins that mediate water transport across the membrane. Water transport is mediated through the osmotic pressure difference between the inside and outside of the cell membrane. Water channel proteins are involved in maintaining the balance of water and electrolytes inside and outside of the cell which is of vital significance [1], Studies have confirmed that aquaporin is an important molecule that maintains the balance and stability of water and electrolytes in the body [1,2].

Recent studies have identified 13 members of the aquaporin (AQP) family (AQP0–AQP12) in mammals. They may also be divided into two major types according to function [3]. The first type, which is permeable only to water, and functions to reabsorb water, includes AQPO, AQP1, AQP2, AQP4, AQP5, AQP6, and AQP8. The second type of aquaporin reabsorbs antidiuretic hormones and includes AQP3, AQP7, and AQP10. These two types of aquaporins can transport not only water, but also glycerol. The function of AQP11 and AQP12 is still unclear. Water channel proteins may be found in the respiratory, digestive, urinary, and cardiovascular system, For each system, in the same organ, tissue, and cell, two or more water channel proteins may be involved in the distribution of water, and work together to regulate water homeostasis. However, the function and effect of the same aquaporins in different cells or systems are not exactly the same, and there may be specific differences.

Studies have confirmed that AQPs 1–4 are involved in the reabsorption of water by renal tubules and play an important role in regulating urinary excretion. Of these, AQP1 is distributed in the acroplasmic and lateral membrane of the proximal convoluted tubule and descending branch of the loop of Henle, where glomerular filtration fluid is reabsorbed [4]. In vivo experiments have shown that AQP1 deficiency reduces the permeability of the proximal convoluted tubules and descending branches of the loop of Henle, reduces liquid reabsorption capacity, and destroys the countercurrent multiplication system [5]. Studies have shown that the expression of AQP1 is primarily regulated by high osmotic pressure in the basolateral region, whereas the induction of the intracellular osmotic gradient is not necessary [5]. AQP2 exists in cytoplasmic vesicles under the luminal membrane of the host cells of the renal collecting duct. It may be phosphorylated and transported to the luminal membrane under the regulation of vasopressin (AVP) and plays an important role in urine concentration [6–8]. AQP3 is distributed throughout the whole collecting duct system from the renal cortex to the renal papilla, and is highly expressed in the base membrane of the collector tube and basal lateral membrane of the collecting duct. AQP4 is primarily expressed in the basal membrane of host cells of the inner medulla and the outer one-third of the inner medulla collecting duct. AQP3 and AQP4 together diffuse water transported into cells by AQP2. Although AQP6, 7, 8, and 11 are distributed in the nephridium, they do not play a major role in renal water transport and urine concentration.

At least eight aquaporins have been reported to be expressed in the digestive system, which include AQP1, AQP3, AQP4, AQP5, AQP8, AQP9, AQP10, and AQP12. AQP1 is widely expressed in capillaries and epithelial cells of small vessels of the digestive system [9]. The expression of AQP5 in human salivary glands is significantly correlated with salivary secretion, plays an important role in bile formation, and may also be involved in the intercellular transport of chylomicrons and water in the pancreatic duct [10]. In addition, AQP1 mediates pancreatic secretion and reabsorption of the fluid in the colon. AQP3 is highly expressed in both the upper and lower digestive tracts. AQP3 is expressed in the basal membrane of the mucosal epithelial cells on the surface of the gastric gland and in the tip of the villi of the small intestine. There is abundant expression in the basal membrane of absorbent epithelial cells in the distal colon and rectum, but not in the cecum or proximal colon. Its physiological function on the epidermis has been studied in AQP3 knockout mice. Experiments show that the hydration of the tissue cuticle and the elasticity of the skin were significantly reduced compared with the control group, thus it was speculated that similar physiological effects occur in the digestive tract. AQP4 is expressed in the basal membrane of gastric parietal cells, but is more abundant in the parietal cells at the base of the gastric fovea. AQP4 is located in the basal membrane of crypt cells in the small intestine and the epithelial cells on the surface of the colon. In a study of wild-type AQP4 knockout mice, transcellular water transport through AQP4 in colonic epithelial tissue contributed to the increase in water permeability under osmotic pressure across the epithelium, but had little effect on colonic fluid secretion or fecal dehydration [11].

In traditional Chinese medicine, it is believed that under normal physiological conditions, the transport, distribution, and excretion of water and liquid is a complex physiological process, which is closely associated with the lungs, spleen, and nephridium, and involves multiple viscera. This is similar to modern research results of ‘aquaporin widely exists in nephridium, lung, and digestive organs, and is distributed in all tissues and organs of the whole body, plays a role in mediating water transmembrane transport, and is the molecular basis for maintaining the balance of water and liquid metabolism in the body.’ Therefore, it may be inferred that the regulation of water metabolism by the lung, spleen, and nephridium occurs through the aquaporins.

Under pathological conditions, the abnormal function of splenic fluid transport and nephridium warm gasification are the most common etiologies and pathologies associated with abnormal fluid metabolism. The main transport of water by the spleen indicates that the spleen functions by absorbing and distributing water and preventing the stagnation of water in the body. If the spleen is deficient in Yang and fails to liquefy water and grain into body fluid, the excess water in the stomach and intestines remains. In addition, if the spleen fails to transfer body fluids to the lungs, nephridia, or other viscera and organs in time, then body stagnation results in phlegm, dampness, or coagulation in the viscera. It may also flow into the intestines or spill over into the skin. Nephridia are the origin of congenital. It is the motivity of organism life activities and also the motivity of gasification. The stomach converts food into nutrients that the body needs and the spleen carries it throughout the body. The lungs distribute water around the body. The small intestine separates what is needed from what is not. These functions depend on the transpiration and gasification of the nephridia to distribute the material throughout the body, whereas the unwanted material is excreted in the urine. The amount of urine excretion is an important mechanism to regulate the balance of water metabolism throughout the body. Through water metabolism, the function of spleen transport and nephridium warm gasification is linked with aquaporin. If the regulation of the spleen and nephridium on water metabolism is dysfunctional, the expression of aquaporin may be abnormal. This may result in abnormal water metabolism causing sputum, urination, dysuria, cough, and asthma.

In recent years, preliminarily studies have confirmed this connection. Many physicians have observed changes in the expression of AQPs in animal models of traditional Chinese medicine (TCM) syndromes, such as lung Qi deficiency, nephridium Yang deficiency, spleen Yang deficiency, and damp-heat syndrome. There are also some reports on the use of Chinese medicine to regulate aquaporin [12,13]. However, few studies have explored the underlying basis of TCM treatment principles and methods from the perspective of aquaporin, and its regulatory mechanism.

In this study, Adriamycin-induced nephropathy rats were used as a research model to study the effects of Wuling Decoction on renal aquaporin, and its regulation. We identified differences in clinical efficacy between the two and further clarified the movement of the spleen, opening and closing of the kidney, and provided a foundation for further study of spleen Yang and nephridium Yang deficiency.

## 2. Materials and methods

### 2.1. Grouping and treatment of rats

SPF male Wistar rats (280 ± 20 g) were purchased from the Chengdu Dashuo Laboratory Animal Co., Ltd. Before the experiment, all animals were acclimated for 3 days in a sterile animal room (24 ± 1.1°C, 60 ± 5% humidity, 12 h/12 h alternating light and dark cycle, withy free access to standard laboratory feed and water. All treatments and procedures in this study were approved by the animal ethics Committee of Guangdong Provincial Hospital of TCM (NO.2016039).

The rats were divided into a blank control group (5 rats), dosing control group (10 rats), Adriamycin nephropathy model group (10 rats), diarrhea group (10 rats), and an Adriamycin nephropathy diarrhea model group (10 rats). Each rat was housed in an independent metabolic cage for 24 h. Rats in the blank group were given normal saline by intragastric administration for 4 days. The control group was given Wuling Decoction which was composed of poria cocos, alisma orientalis, poria pori, cassia twig, and atractylodes adactylies. It was weighed in a proportion of 3:5:3:2:3 and boiled in eight volumes of water four times. The liquid was strained and set aside. Six volumes of water were added and the mixture was boiled three times. The filtrate was mixed and concentrated to 2 kg/L at 80 ℃ followed by pressure cooker sterilization and storage at 4℃. The rats in the Adriamycin nephropathy model group were randomly divided into two groups. Group A was given normal saline by intragastric administration for 4 days, whereas group B was given Wuling Decoction by intragastric administration for 4 days. Rats in the diarrhea group were randomly divided into group A and group B. Group A was given normal saline intragastrically for 4 days, whereas group B was given Wuling Decoction intragastrically for 4 days. Rats in the Adriamycin nephropathy diarrhea group were randomly divided into a group A and B. Group A was given normal saline by intragastric administration for 4 days and group B was given Wuling Decoction by intragastric administration for 4 days. After drug treatment, the animals in each group were sacrificed, abdomen-section was performed, and the middle segment of the ileum and renal tissue samples were harvested.

The rat model of Adriamycin nephropathy was established according to the literature [14]. A solution of Adriamycin was injected into the tail vein at a dose of 7.5 mg/kg. After injection, the needle was removed quickly and sterile gauze was used to press the pinhole to prevent extravasation of drug solution. Urine was collected after injection at day 14 over a 24 -h period. Once the quantity of urine protein for 24 h exceeded 100 mg, the model was considered to be successfully generated.

Diarrhea rat model: The rat diarrhea model was established based on a published report [15]. An 8% Senna leaf decoction was gavaged daily at 2 ml/animal twice a day. Fasting and free drinking water was provided 12 h. The rats were housed in single cages with filter paper and barbed wire at the bottom of the cages. After continuous modeling for 7 days, feces within 5 hours were collected, and the diarrhea index was used to determine the success of the model.

Adriamycin nephropathy diarrhea rat model: The Adriamycin nephropathy model was established by injecting Adriamycin solution (7.5 mg/kg) into the tail vein. After establish the model, the Adriamycin nephropathy diarrhea rat model was generated by intragastric administration of 8% Senna leaf decoction for 7 days.

Determination of diarrhea index: The diarrhea index = loose stool rate (ratio of the number of loose stool per animal to the total number of loose stool) × loose stool grade (rated by the size of the stain area formed by loose stool contamination on the filter paper). It was categorized into four levels: Grade 1 refers to a pollution diameter less than 1 cm; Grade 2 refers to pollution diameter of 1–1.9 cm; Grade 3 refers to pollution diameter of 2–3 cm; and Grade 4 refers to pollution diameter of greater than 3 cm.

### 2.2. Urinary AQP2 and cAMP detection

Each rat was placed into an independent metabolic cage and urine was collected for 24 h. After the urine volume was measured, the supernatant was collected by centrifugation at 2000 rpm for 15 min, and stored at −20℃ for measurement. Urinary AQP2 was detected using an AQP2 ELISA kit (Solarbio).

Diluted serum or urine was added to the enzyme plate. The negative control and positive control were added into the blank wells. The plate was covered and incubated for 30 min at room temperature. The solution was discarded and the plate was washed with detergent and patted dry with absorbent paper. HRP-conjugated antibody was added and the plate was incubated at room temperature for 30 min. After washing and drying, substrate solution A was added to substrate solution B (1:1) and the mixture was applied to the wells. The OD450 and OD630 were measured using a microplate reader.

The cAMP assay was performed using a cAMP assay kit (Wuhan Nustra Biotechnology Co., LTD). A cAMP standard curve was prepared and the concentration of each sample was determined.

### 2.3. Plasma ADH, AVP, cAMP and tissue cAMP were detected

Serum ADH was determined using a antidiuretic hormone kit (Nanjing Yifeixue Biotechnology Co., LTD) and double-antibody sandwich ABC-ELISA method. The coated solution was diluted with anti-ADH-IgG antibody (Nanjing Yifeixue Biotechnology Co., LTD), and added to the HRP-labeled plate, which was covered with the membrane plate, and incubated at 4℃ for 24 h. After washing four times with detergent and drying with absorbent paper, serum at different dilution ratios was added to each well, and the positive and negative control serum was added simultaneously. The serum was placed at room temperature for 30 min. The solution in the well was discarded, washed, and dried with absorbent paper. Diluted HRP- and anti-ADH IgG antibody was added to each well and the plate was incubated at room temperature for 30 min. The solution was discarded, washed, and dried with absorbent paper. A substrate solution was added to each well, placed in the dark for 30 min, and 0.05 ml of 2 M H_2_SO_4_ was added to stop the reaction. OD450 and OD630 were measured with a microplate reader. Serum AVP and tissue homogenate cAMP were determined by radioimmunoassay kits (Beckman Courter Life Center, USA).

### 2.4. Tissue localization of AQP expression as detected by immunohistochemistry

An immunohistochemical method was used to detect the tissue localization of AQP. Colon and nephridium tissue samples were collected, fixed with 10% neutral formaldehyde, and immunohistochemical analysis was performed. The SABC method was used. Paraffin sections (2 μm) were dewaxed, hydrated, antigenic repair was performed, endogenous peroxidase activity was blocked by 3% H_2_O_2_, and rabbit anti-AQP two antibody (1:100) (Merck) was added at 4ʹ overnight. The slides were washed with PBS 3 times for 5 min each. Biotin-labeled secondary antibody (Merck) was added and incubated for 20 min followed by washing with PBS 3 times. SABC reagent was added (Merck) and incubated for 20 min followed by washing with PBS 3 times. A drop of horseradish peroxidase substrate was added for color development. PBS was used as negative control. The image was analyzed using the MIASPRO image analysis system.

### 2.5. AQP expression as measured by RT-PCR

Collected colon and nephridium tissue samples were frozen in liquid nitrogen and fully homogenized at low temperature. Total RNA was extracted with Trizol reagent and cDNA was synthesized in a 20 μl reverse transcription reaction. The cDNA (5 μl) was used as template for RT-PCR. The PCR reactions were set up according to the TAKARA kit instructions. PCR melt curves were assessed and gene expression was quantified. The primers used were 5ʹ-CAGCTCGAAGGAAGGAGACA-3ʹ and 5ʹ-GCATTGGCACCCTGGTTCA-3ʹ for mouse AQP2, and 5ʹ-GCCAAGAGGGTCATCATCTC-3ʹ and 5ʹ-CCTTCCACAATGCCAAAGTT-3ʹ for mouse GAPDH. GAPDH was used as an internal reference. The results were analyzed with ABI 7500 Real Time System SDS software. Gene expression was calculated using the ΔΔct method.

### 2.6. AQPs protein expression detected by western blot analysis

Colon and nephridium tissue samples were collected, placed in a tissue homogenizer, and precooled RIPA buffer and protease inhibitors were added. The tissues were homogenized with an ultrasonic crushing machine, and centrifuged at 12,000 rpm at 4ʹ for 20 min. The BCA method was used to measure protein concentration. The concentration of the samples was normalized by adding RIPA buffer. Next, 2× SDS sample buffer was mixed with the protein samples, and denatured at 95℃ for 5 min. SDS-PAGE was done using 20 μg of protein sample. The separated proteins were transferred to PVDF membranes and placed in blocking solution at 37ʹ for 1 h. The primary antibody was incubated with the membrane at 4ʹ overnight. Membranes were washed with TBST 3 times for 15 min and secondary antibody (1:1000/1:2000) was added for 1 h at room temperature. The membranes were washed with TBST 3 times for 15 min. After development, the intensity of each band was determined using image analysis software.

### 2.7. Statistical analysis

Garphpad Prism 8.0 (Graphpad Software, Inc, La Jolla, CA, USA) was used for statistical analysis. All results were expressed as the mean ± SD. The Image J software processing system was used to analyze WB results and calculate the optical density values. Differences between experimental groups were assessed by t-test or one-way analysis of variance and t-test of significance. P < 0.05 was considered statistically significant.

## 3. Experimental results

### 3.1. Wuling Decoction ameliorates diuresis and anti-diarrhea by regulating the level of AQP2

**Figure 1.**
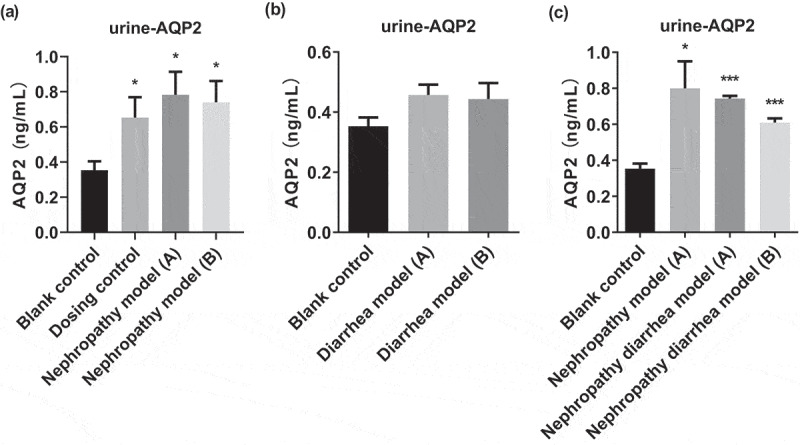
Urine aquaporin 2 (AQP2) protein content increases in each group (a) No significant change was observed in the diarrhea model group, (b) Urine AQP2 content increased in each group. There was no significant change in the model groups after Wuling Decoction treatment (c). *P < 0.05, **P < 0.01, ***P < 0.001 vs. Control group.

The content of AQP2 in the urine of rats in each group was measured. The results showed that the urine AQP2 content was significantly increased in the dosing control group, nephropathy model group, and nephrotic diarrhea group. However, the increase of urine AQP2 in the nephrotic model group and nephrotic diarrhea group was more pronounced, suggesting that kidney injury after Adriamycin administration resulted in a significant increase in urine AQP2. In the diarrhea model group, there was no significant change in urine AQP2 content. After Wuling Decoction treatment, the content of AQP2 in the urine did not change significantly (Figure 1A, 1B). This suggests that the body reduces water reabsorption by up-regulating AQP2 expression, thus achieving a diuretic effect. Wuling Decoction down-regulated AQP2 expression in rats with nephropathy and diarrhea (Figure 1C). Based on the above changes of urine AQP2 levels in each group, Wuling Decoction may reduce renal damage in nephropathy and diarrhea model rats. It has a mild diuretic effect and relieves diarrhea by modulating the change of AQP2 levels in the urine.

### 3.2. Wuling Decoction affects water absorption by regulating the levels of ADH and AVP

**Figure 2.**
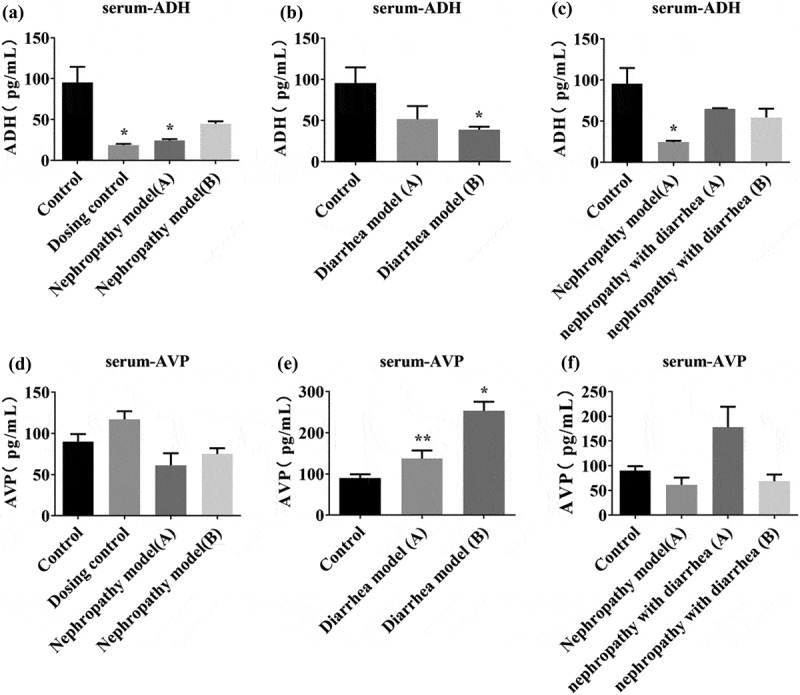
Serum antidiuretic hormone (ADH) and arginine vasopressin (AVP) expression levels in each group. Serum ADH levels in the control and nephrotic model group A were significantly decreased. After Wuling Decoction treatment, serum ADH increased in nephropathy model group A. In the Diarrhea model group, ADH levels in group A and B were lower compared with those in the control group (b). The level of serum ADH decreased in the Nephropathy with diarrhea model group, however, Wuling Decoction had no significant effect after treatment (c). After Wuling Decoction treatment, the level of AVP in the control group increased, and the level of serum AVP in Nephropathy model groups A and B decreased (d). The level of AVP in the Diarrhea model group A was significantly increased, whereas the level of AVP in Diarrhea model group B was increased after Wuling Decoction treatment (e). Following Wuling Decoction treatment, serum AVP levels of Nephropathy with diarrhea model group A were significantly increased, whereas that of Nephropathy with diarrhea model group B were significantly decreased (F). *P < 0.05, **P < 0.01 vs. Control group.

Serum ADH and AVP levels were measured in rats. The results showed that the level of serum ADH in the control group and nephropathy model group A decreased significantly. After treatment with Wuling Decoction, serum ADH levels in the nephropathy model group increased significantly (Figure 2A). In the diarrhea model group, ADH levels in group A were lower compared with that in the control group, and ADH levels in group B were significantly lower (Figure 2B). Although serum ADH levels in the nephropathy with diarrhea model group decreased, Wuling Decoction exhibited no effect (Figure 2C). The level of AVP in the dosing control group increased, the level of serum AVP in the Nephropathy model group A decreased, and the level of serum AVP in the Nephropathy model group B increased following Wuling Decoction treatment (Figure 2D). The level of AVP in Diarrhea model group A increased significantly and the levels in diarrhea model group B increased after Wuling Decoction treatment (Figure 2E). Serum AVP levels in nephropathy with diarrhea model group A was significantly increased, whereas that of nephropathy with diarrhea group B was significantly decreased after treatment with Wuling Decoction (Figure 2F).

### 3.3. Wuling Decoction decreases the level of cAMP in tissues, thus reducing the absorption of AQP2 to water

**Figure 3.**
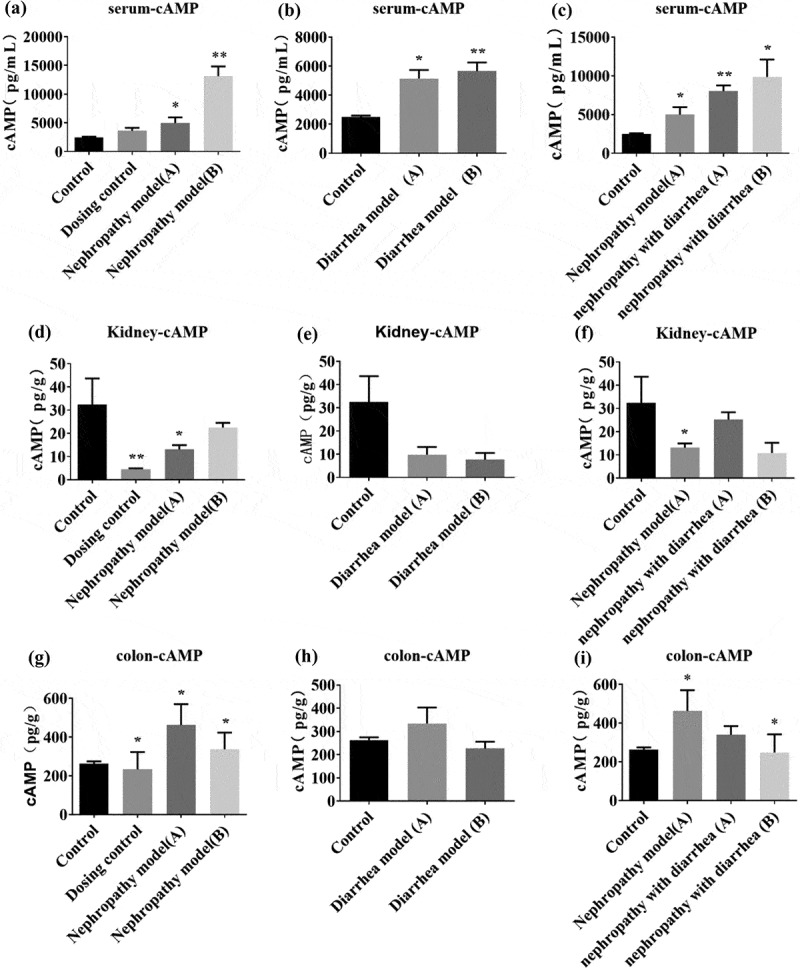
Serum, nephridium, and intestinal cyclic adenosine monophosphate (cAMP) levels. Serum cAMP was up-regulated in all groups and the level of cAMP increased after Wuling Decoction treatment (A). cAMP levels in the diarrhea group increased, but no significant change was evident after treatment (B). cAMP levels increased in the Nephrotic diarrhea group, and then increased after treatment (C). Kidney tissue cAMP levels in the Nephropathy group decreased significantly, but increased after treatment (D). cAMP levels in the Diarrhea group decreased significantly, but no significant change was evident after treatment (E). cAMP did not change significantly in the Nephrotic diarrhea group, but decreased after treatment (F). Intestinal tissue cAMP levels increased in the Nephropathy group, but decreased after treatment (G). cAMP levels in the Diarrhea group exhibited no significant change after treatment (H). cAMP levels in the Nephrotic diarrhea group showed no significant change, but decreased after treatment (I). (*P < 0.05, **P < 0.01 vs. Control group.).

cAMP levels were measured in the serum, nephridium, and colon. Serum cAMP was up-regulated in all groups, and the level of cAMP increased again after Wuling Decoction treatment (Figure 3A, 3B, 3C). In nephridia, cAMP levels in both the dosing control group and nephropathy model group A decreased, whereas cAMP levels in nephropathy model group B increased slightly after Wuling Tang treatment (Figure 3D). The cAMP levels in the nephridium of the diarrhea model group decreased and there was no significant change before and after Wuling Decoction treatment (Figure 3E). In nephridia, cAMP levels were decreased in the nephropathy with diarrhea group A, and the level of cAMP was lower after Wuling Decoction treatment (Figure 3F). In the colon, cAMP levels were decreased in the dosing control group and significantly increased in the nephropathy model group, which was down-regulated after Wuling Decoction treatment (Figure 3G). The level of cAMP in the diarrhea model group increased, but decreased after Wuling Decoction treatment (Figure 3H). cAMP levels in the Adriamycin nephropathy diarrhea group increased and decreased significantly after Wuling Decoction treatment (Figure 3I).

### 3.4. Wuling Decoction inhibited the expression of AQP2 in renal tissues and promoted the expression of AQP2 in intestinal tissues

**Figure 4.**
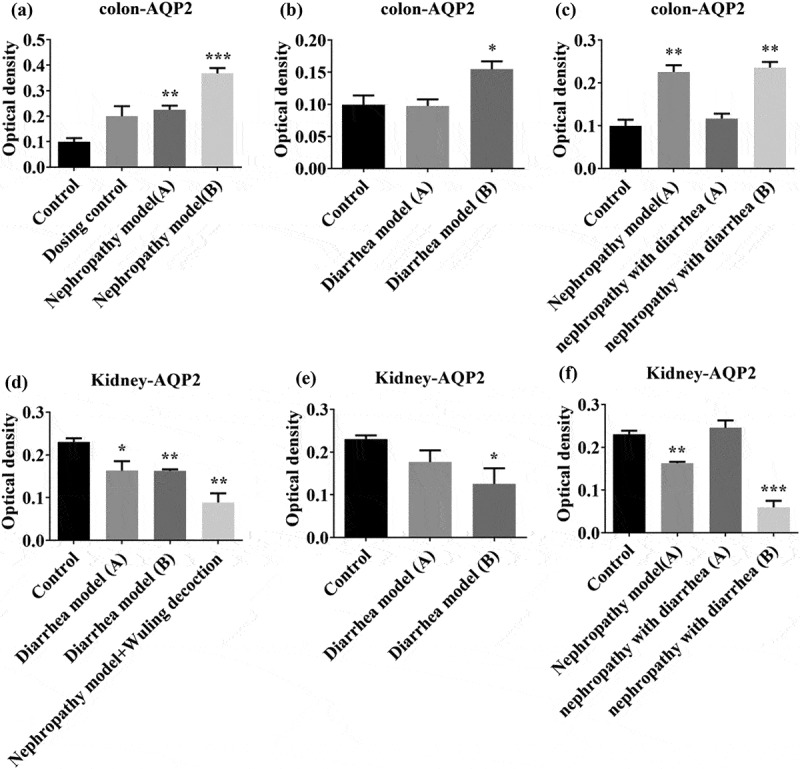
Expression of aquaporin 2 (AQP2) in the colon and nephridium. Intestinal tissue AQP2 expression was increased in the Nephropathy group and again after treatment (A). There was no significant change in the Diarrhea group, however, AQP2 expression increased after treatment (B). The expression of AQP2 increased after treatment (C). Renal tissue AQP2 expression was decreased in the Nephropathy group, and again decreased after treatment (D). There was no significant change in the Diarrhea group, but the expression was decreased after treatment (E). There was no significant change in the Nephropathy and Diarrhea group, whereas the expression decreased after treatment (F). *P < 0.05, **P < 0.01 vs. Control group.

We assessed AQP2 expression in nephridia and colon tissues by immunohistochemistry. The results showed that in the colon, AQP2 levels were increased in the dosing control group and nephropathy model group A. Following Wuling Decoction treatment, AQP2 levels were increased in the nephropathy model group B (Figure 4A). There was no significant change in AQP2 levels in nephropathy model group A, however, after Wuling Decoction treatment, AQP2 levels in nephropathy model group B were significantly increased (Figure, 4B). There was no significant change in AQP2 levels in nephropathy with diarrhea group A, but after Wuling Decoction treatment, AQP2 levels in nephropathy with diarrhea group B were increased significantly (Figure 4C). In the nephridia, AQP2 levels were significantly decreased in both the administration group and nephropathy group A, and again decreased in nephropathy group B after Wuling Decoction treatment (Figure 4D). AQP2 levels in diarrhea model group A decreased slightly after Wuling Decoction treatment, whereas AQP2 levels in group B decreased significantly (Figure 4E). There was no significant change in AQP2 levels in nephropathy with diarrhea group A, whereas it was significantly reduced in group B after Wuling Decoction treatment (Figure 4F).

### 3.5. Wuling Decoction promotes AQP2 expression in the Nephropathy model group and inhibits AQP2 expression in the Diarrhea group

**Figure 5.**
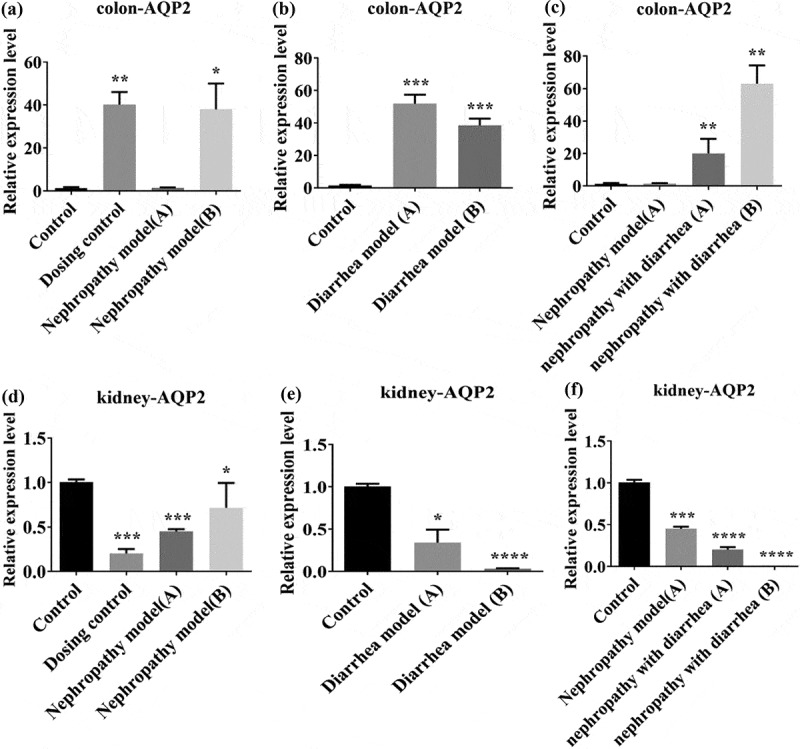
Intestinal tissue: Expression of Aquaporin 2 (AQP2) was not significantly altered in the Nephropathy group, whereas it was increased after treatment with Wuling Decoction (A). The expression of AQP2 increased in the Diarrhea group and decreased after treatment (B). The expression of AQP2 increased in the Adriamycin nephropathy diarrhea group and increased after Wuling Decoction treatment (C). Renal tissue: AQP2 expression was significantly decreased in the Nephropathy group and increased after treatment (D). The expression of AQP2 decreased in the Diarrhea group and again after treatment (E). The expression of AQP2 decreased in the Adriamycin nephropathy diarrhea group and decreased after treatment (F). (*P < 0.05, **P < 0.01, ***P < 0.001, ****P < 0.0001 vs. Control group.).

**Figure 6.**
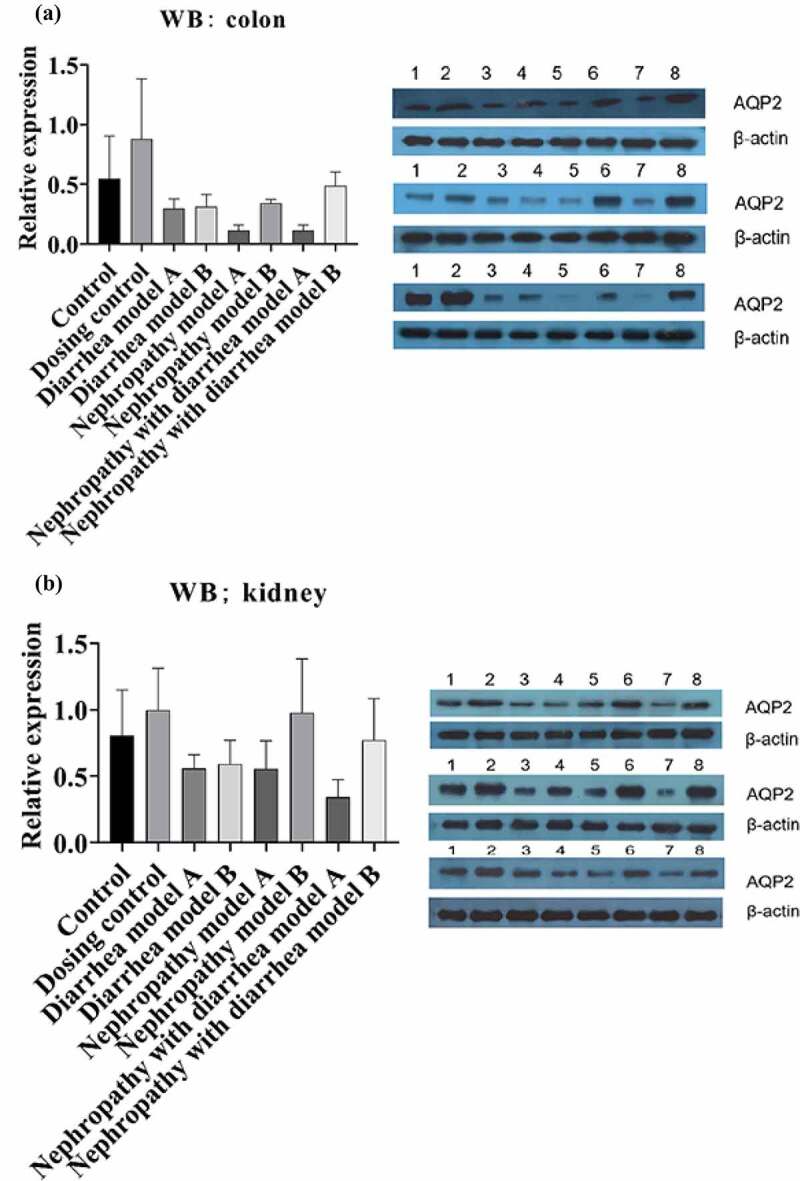
Western blot analysis showing aquaporin 2 (AQP2) protein expression in intestinal tissues of each group (A). Western blot analysis showing AQP2 protein expression in renal tissues of each group (B). 1: Control; 2: Dosing control; 3: Diarrhea model A; 4: Diarrhea model B; 5: Nephropathy model A; 6: Nephropathy model B; 7: Nephropathy with diarrhea model A; 8: Nephropathy with diarrhea model B.

Quantitative PCR and WB were used to detect AQP2 gene expression in intestinal and renal tissues. The qPCR results showed that in the colon, AQP2 levels in the dosing control group increased significantly, whereas there was no significant change in nephropathy model group A. After Wuling Decoction treatment, AQP2 expression levels in group B were increased significantly (Figure 5A). The AQP2 levels in diarrhea model group A increased significantly, whereas that of group B decreased after Wuling Decoction treatment (Figure 5B). The AQP2 levels in nephropathy with diarrhea model group A increased significantly, whereas that of group B increased significantly following treatment with Wuling Decoction (Figure 5C). In nephridium, the AQP2 levels in the dosing control group and nephropathy model group A decreased significantly. After Wuling Decoction treatment, AQP2 levels in nephropathy group B increased (Figure 5D), whereas AQP2 levels in diarrhea model group A decreased significantly. After Wuling Decoction treatment, AQP2 levels in group B decreased significantly (Figure 5E). AQP2 levels were significantly decreased in the sick diarrhea model group, and decreased in group B after Wuling Decoction treatment (Figure 5F). WB analysis of the colon revealed that AQP2 expression increased in the dosing control group, but decreased in the other model groups. There was no significant change in the nephropathy model after Wuling Tang treatment, however, an increase in the diarrhea model group and nephropathy with diarrhea group was observed after Wuling Tang treatment (Figure 6A). WB results of the nephridium samples showed that AQP2 levels increased in the dosing control group, but decreased in all model groups. After Wuling Decoction treatment, the AQP2 levels increased (Figure 6A).

## 4. Discussion

Wuling Decoction has the effect of warming Yang, removing Qi, reducing dampness, and promoting water. It is primarily used for bladder Qi maladies, urine problems caused by water moisture cohesion, edema, abdominal distension, nausea and diarrhea, and thirst. Studies have shown that Wuling Decoction supplemented with Tianma Jingteng Decoction improves blood pressure and cardiac function in patients with hypertension [16]. Moreover, Wuling Decoction also plays a role in the treatment of chronic heart failure [17]. Studies have found that Wuling Decoction combined with ventriculo-peritoneal shunt is superior to ventriculo-peritoneal shunt alone for the treatment of idiopathic atmospheric hydrocephalus [18]. AQP2 is primarily expressed in collecting duct epithelial cells and plays an important role in urine concentration. Studies have shown that changes in AQPs occur in many diseases. In chronic obstructive pulmonary disease, AQP5 expression is associated with the progression of COPD, and affects lung function [19–21]. In esophageal cancer, the expression levels of AQP3 and AQP5 are significantly higher compared with those in adjacent normal tissues [22]. AQP4 and AQP5 are abnormally expressed in gastric cancer [23]. AQPs are also targets for many diseases, such as pyelonephritis and IgA nephropathy, in which abnormal levels of AQP2 can be detected in the urine, suggesting that AQP2 may be a therapeutic target for this disease [24,25]. In renal fibrosis, inhibiting EMT can ameliorate the loss of AQP1, indicating that AQP1 is involved in the EMT process [26].

In the present study, Wuling Decoction was evaluated in Adriamycin-induced nephropathy rats. The results indicated that Wuling Decoction affects water absorption by excreting AQP2 from the body. Wuling Decoction regulates the content of ADH and AVP in serum to regulate water metabolism. AVP is secreted when the osmotic pressure of the body increases to increase the reabsorption of water, so that the body maintains normal osmotic pressure. ADH has a similar effect on AVP and AQP2 is the only aquaporin sensitive to ADH [27]. When ADH or AVP binds to cell receptors, it activates adenylate cyclase, promotes the production of large amounts of cAMP in the body, and eventually leads to the activation of AQP2 to concentrate urine, and reabsorb water to maintain osmotic pressure [28].

Wuling Decoction can down-regulate the level of cAMP in serum and tissues to regulate the absorption of water. Studies have shown that cAMP can phosphorylate serine via PKA to increase the permeability of the cell membrane to water and increase water absorption [29,30]. Overall, Wuling Decoction reduces AQP2 content in renal tissue, inhibits the expression of AQP2 in renal tissue, reduces the level of cAMP in serum, reduces the reabsorption of AQP2 to water, and causes a diuretic effect. By increasing the expression of AQP2 in intestinal tissue, Wuling Decoction increases the reabsorption of intestinal water, to alleviate anti-diarrhea.

## 5. Conclusion

We confirmed the process of “relieving urine to solidify stool” by Wuling Decoction can ameliorate diuresis and diarrhea by expelling AQP2 from the body, inhibiting the expression of AVP, ADH and cAMP, inhibiting the expression of AQP2 in renal tissues, and promoting the expression of AQP2 in intestinal tissues. The results of our study provide a molecular biological basis for a deeper understanding of Chinese medicine.

## Data Availability

The data used to support the findings of this study are included within the article.
